# Evaluation and identification of morphological characters suitable for delimitation of *Taraxacum* species distributed in northeastern China

**DOI:** 10.1002/fsn3.2896

**Published:** 2022-04-22

**Authors:** Wu Jie, Liu Qun, Haitao Cheng, Wei Ning, Wei Cao

**Affiliations:** ^1^ School of Public Health Shenyang Medical University Shenyang China; ^2^ Institute of Botany Jiangsu Province and Chinese Academy of Sciences (Nanjing Botanical Garden Mem. Sun Yat‐Sen) Nanjing China; ^3^ 58575 School of Traditional Chinese Materia Medica Shenyang Pharmaceutical University Shenyang China; ^4^ 98428 Shenyang Agricultural University Shenyang China; ^5^ Institute of Applied Ecology Chinese Academy of Sciences Shenyang China

**Keywords:** cluster analysis, clustering analysis, morphological characteristics, principal component analysis, *Taraxacum*

## Abstract

*Taraxacum* germplasm resources in northeastern China are not current and do not accurately reflect the actual distribution of the species. The objective of this study was to investigate the morphological traits of *Taraxacum* species distributed in northeastern China and identify those that will facilitate their classification in this region. Leaf, flower, and achene characteristics of 18 species were used for morphological classification. Scanning electron microscopy (SEM) was used to examine pollen morphology. Internal transcribed spacer (ITS) sequences were analyzed to determine sequence differences among the species and their utility in delimitation. Taxa were classified into groups based on their morphology. The ITS sequence analysis supported the taxon classification, but the genetic distances among the taxa did not reflect morphological differences. Phylogenetic analysis was used to divide the 18 species into three groups. Group I: *T*. *coreanum* (which has white flowers). Group Ⅱ: *T. heterolepis*, *T. sinomongolicum*, *T*. *variegatum*, *T. asiaticum* var*. lonchophyllum*, *T. falcilobum*, *T. brassicaefolium*, and *T. erythropodium* (outer involucre bracts, narrow membranous or nonmembranous). Group Ⅲ: *T*. *formosanum*, *T*. *liaotungense*, *T*. *mongolicum*, *T. borealisinense*, *T*. *ohwianum*, *T. platypecidum*, *T*. *urbanum*, *T. antungense*, *T*. *asiaticum*, and *T. junpeianum* (outer involucre bracts, broad membranous). The main taxonomic characteristics of *Taraxacum* floral organs and achene morphology.

## Graphical abstract

The study aimed to categorize 18 *Taraxacum* taxa from northeastern China based on DNA data and cluster analysis of morphological characteristics and test whether the latter supports the molecular‐based phylogeny.

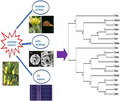

## 1 INTRODUCTION

The genus *Taraxacum* F. H. Wigg in the aster family (*Compositae*) includes about 2500 species, mainly in the Arctic and temperate zones of the Northern Hemisphere; species diversity is high in the mountains of Eurasia, and a few species are found in temperate regions of the Southern Hemisphere (Ge et al., 2012). The interaction between genetic variation and the environment produces morphological variation in *Taraxacum* and the formation of agamospermous complexes (Lin & Xuejun, 1999). Many *Taraxacum* species are apomictic, and some asexual genotypes may be more predisposed to genomic changes than other asexual genotypes (Aquaro et al., 2008; Li, 2004). The various ploidy levels and intraspecific morphological variations of *Taraxacum* species complicate their classification (Cortés et al., 2014).

Previous studies have employed morphological characteristics related to achene shape and color, overall achene length, achene beak length, leaf shape, leaf length, and leaf color to distinguish between *Taraxacum* species (Hidayat et al. (2016)). The taxonomic significance of seed (achene) structure has been highlighted by multiple researchers. For example, Ullah et al. (2021) investigated the ultrastructure of fruit and seed surface morphology among populations of the alpine *Rosa sericea* complex (Rosaceae) using scanning electron microscopy (SEM). Morphological and foliar characteristics are important tools for the identification of different plant groups, and they have been examined under light microscope (LM) and SEM to determine the taxonomic implications of the leaf epidermal anatomy of selected taxa of Scrophulariaceae from Pakistan (Ullah et al., 2020). Pollen morphology has assumed great significance in plant taxonomy. SEM has been used to evaluate the pollen diversity of the genus *Sophora* (Fabaceae) and its taxonomic significance (Liao et al., 2021).

The internal transcribed spacer (ITS) region of nuclear ribosomal DNA consists of internal transcribed spacer 1 (ITS‐1), the 5.8S, and internal transcribed spacer 2 (ITS‐2). This region has been widely used in the molecular phylogenetic analyses of many plant taxa, such as *Asarum sieboldii* Miq., *Withania somnifera* (L.) Dunal., *Convolvulus prostrates* Linn, and *Evolvulus alsinoides* (L.) L. var (Kim et al., 2014; Lee et al., 2013). In addition, a conserved 14 bp motif (5′‐GAA TTG CAG AAT CC‐3′) was found in the 5.8S gene, which is useful to differentiate between flowering plants and other plant groups (Martinez et al., 2015; Xiao et al., 2021). Kirschner revised the classification of dandelions in Central Asia by using color digital pictures to compare the characteristics seen in these with the known classification of dandelions, which is an important advancement in the classification of dandelions in Central Asia (Jan, 2016). In this study, we aimed to categorize 18 *Taraxacum* taxa from northeastern China based on DNA data and cluster analysis of morphological characteristics, and to evaluate whether the latter supports molecular‐based phylogeny.

## 2 MATERIALS AND METHODS

### 2.1 Plant materials and growing conditions

**FIGURE 1:**
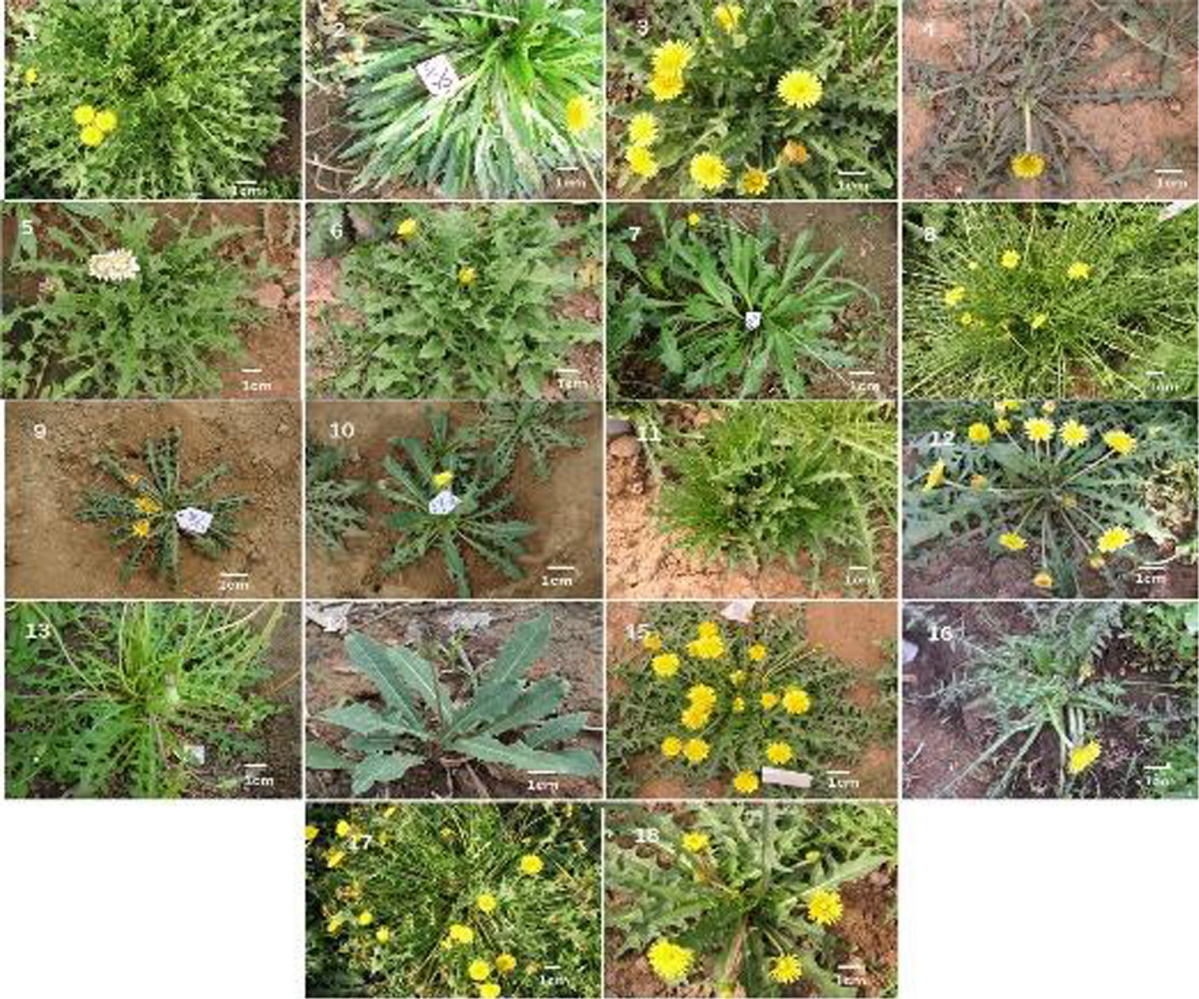
Pictures of the 18 taxa of northeast China native *Taraxacum* species

The flora of northeastern China harbors 18 native *Taraxacum* taxa, which are defined based on the leaf margin, flower color, shape and texture of the outer bracts, shape of the inner bracts, achene length, and other characteristics. Their distribution in this region is shown in Figure 1. Eighteen taxa were collected from their natural habitats from April to October each year from 2011 to 2019; more than 30 individuals were collected for each taxon (Table 1 and Figure 1). The classification and nomenclature of *Taraxacum* species were mainly done in accordance with Flora Plantarum Herbacearum Chinae Boreali‐Orientalis. Voucher specimens were deposited in the Liaoning Medicinal Herbarium at Shenyang Agricultural University at the Ex situ Conservation Garden Evaluation Centre of Wild Vegetable Germplasm in Northeast China, Ministry of Agriculture. Individual plants were grown in a greenhouse (41°82′N, 123°56′E) at an altitude of 80 m above sea level. Plants were collected at anthesis, processed in herbarium specimens, and deposited at the Institute of Applied Ecology, Chinese Academy of Sciences. Five individuals from each taxon were randomly selected, and their morphological characteristics were assessed.

**TABLE 1:** Collection locations of the 18 *Taraxacum* species in Northeast China

No.	Latin	Population name	Origin	Voucher
1	*T. antungense* Kitag.	Dandong dandelion	Dandong City, Liaoning province	001(SYAU)
2	*T. asiaticum* Dahlst.	Yazhou dandelion	Jilin City, Jilin Province	002(SYAU)
3	*T. variegatum* Kitag.	Banye dandelion	Dandong City, Liaoning Province	003(SYAU)
4	*T. mongolicum* Hand.‐Mazz.	Menggu dandelion	Chifeng City, Neimengu province	004(SYAU)
5	*T. coreanum* Nakai.	Chaoxian dandelion	Shenyang City, Liaoning province	005(SYAU)
6	*T. ohwianum* Kitag.	Dongbei dandelion	Heihe City, Heilongjiang province	006(SYAU)
7	*T. urbanum* Kitag.	Juanbao dandelion	Changchun City, Jilin province	007(SYAU)
8	*T. asiaticum var. lonchophyllum* Kitag.	Xiajipian dandelion	Chaoyang City, Liaoning province	008(SYAU)
9	*T. formosanum* Kitam.	Taiwan dandelion	Tongliao City, Neimengu province	009(SYAU)
10	*T. liaotungense* Kitag.	Liaodong dandelion	Songyuan City, Jilin province	010(SYAU)
11	*T. sinomongolicum* Kitag.	Tujian dandelion	Yichun City, Heilongjiang province	011(SYAU)
12	*T. heterolepis Nakai et Koidz. ex* Kitag.	Yibao dandelion	Siping City, Jilin province	012(SYAU)
13	*T. brassicae folium* Kitag.	Jieye dandelion	Anshan City, Liaoning province	013(SYAU)
14	*T. platypecidum* Diels.	Baiyuan dandelion	Tongliao City, Neimengu province	014(SYAU)
15	*T. falcilobum* Kitag.	Xingan dandelion	Yichun City, Heilongjiang province	015(SYAU)
16	*T. borealisinense* Kitam.	Hua dandelion	Songyuan City, Jilin province	016(SYAU)
17	*T. junpeianum* Kitam.	Changchun dandelion	Shenyang City, Liaoning province	017(SYAU)
18	*T. erythopodium* Kitag.	Honggeng dandelion	Xinganmeng City, Neimengu province	018(SYAU)

Note

Resource: investigated on location by authors.

### 2.2 Measurement of morphological characteristics

Vegetative and reproductive morphological traits that distinguish *Taraxacum* species were selected based on the treatment of the genus. The plant height was measured from the highest vertical point (stem or leaf apex) to the root tip. The largest leaves with a normal morphological appearance were selected for measurements. The leaf length was measured from the lamina tip to its base, excluding the petiole. The leaf width was measured across the widest point. The leaf thickness was measured across the thickest part. The shape, size, color, and ornamentation characteristics of achenes (e.g., shape and amount of ornamentation) were observed on the fruit wall. The shape and size of the coracoid base and the transition of the fruit body to the coracoid base were constricted suddenly or contracted gradually. The beak thickness and length, color of the crested hair, and beak length ratio (BL/AL: beak base length to achene length) were also measured.

Pollen morphology was examined using SEM (KYKY 10000B; Science Instrument Company, Beijing, China) in the natural mode; additionally, all disks were observed using SEM (KYKY SBC‐12 ion sputter coater). The pollen size (polar length [*P*], equatorial diameter [*E*], and *P*/*E* ratio) was calculated using Motic Images Advanced 3.2 software (Motic, Hong Kong, China). Size measurements were based on 20 pollen grains. The pollen grains underwent acetolysis and were mounted in glycerin jelly. Mature achenes were collected from each species two months after flowering and photographed with a camera (Moficam 2206; Olympus, Japan) attached to a light microscope.

### 2.3 ITS sequence analysis

Genomic DNA from *Taraxacum* leaf tissue was extracted and purified using a modified cetyltrimethylammonium bromide (CTAB) method. The quality and quantity of the isolated DNA were verified using agarose gel electrophoresis. The universal forward and reverse primers, ITS‐*F* (5′‐AGG TGA ACC TGC GGA AGG ATC ATTG‐3′) and ITS‐R (5′‐CTT CTC CTC CGC TTA TTG ATA TGCT‐3′), were used to amplify the ITS region. A total of 25 ng genomic DNA and 5 pmol of each primer were used in the reaction that included 1 μmol·L^−1^ of primer, 2.0 mmol·L^−1^ MgCl_2_, 1.6 mmol·L^−1^ of deoxyribonucleoside triphosphate (dNTP), 0.4 mmol·L^−1^ of Taq polymerase, and 50 ng of template DNA. Polymerase chain reaction (PCR) was carried out in a thermocycler (Bio‐Rad PTC‐100 PCR) programmed as follows: 95°C for 5 min; 35 cycles of 95°C for 30 s, 60°C for 30 s, and 72°C for 1 min, with a final extension at 72°C for 10 min. Amplicons were purified using a PCR Cleanup Kit (Axygen Scientific, Inc.) and sequenced by Sangong Biotech (Shanghai, China). To determine the ITS sequence of dandelion more completely and effectively, sequences were checked against other *Taraxacum* ITS sequences in GenBank using the Basic Local Alignment Search Tool to construct a database model of molecular biomolecule (BLAST; National Center for Biotechnology Information).

### 2.4 Data analysis

Data for 32 qualitative and quantitative morphological characteristics of the 18 taxa were subjected to analysis of variance (ANOVA) using SPSS v.21.0 at significance levels of *p* ≤ .01 and *p* ≤ .05, principal component analysis (PCA), and cluster analysis.

The obtained sequences of the ITS region were aligned using Clustal X and adjusted manually. Phylogenetic tree reconstruction based on the parsimony method was performed using PAUP* version 4.0b10. The insertions and deletions were treated as missing data. Clades’ support was evaluated using the bootstrap analysis with 1000 replicates. The number of steps, consistency indices, and retention indices were calculated using the TREE SCORE command in PAUP*.

## 3 RESULTS

### 3.1 Growth characteristics

**FIGURE 2:**
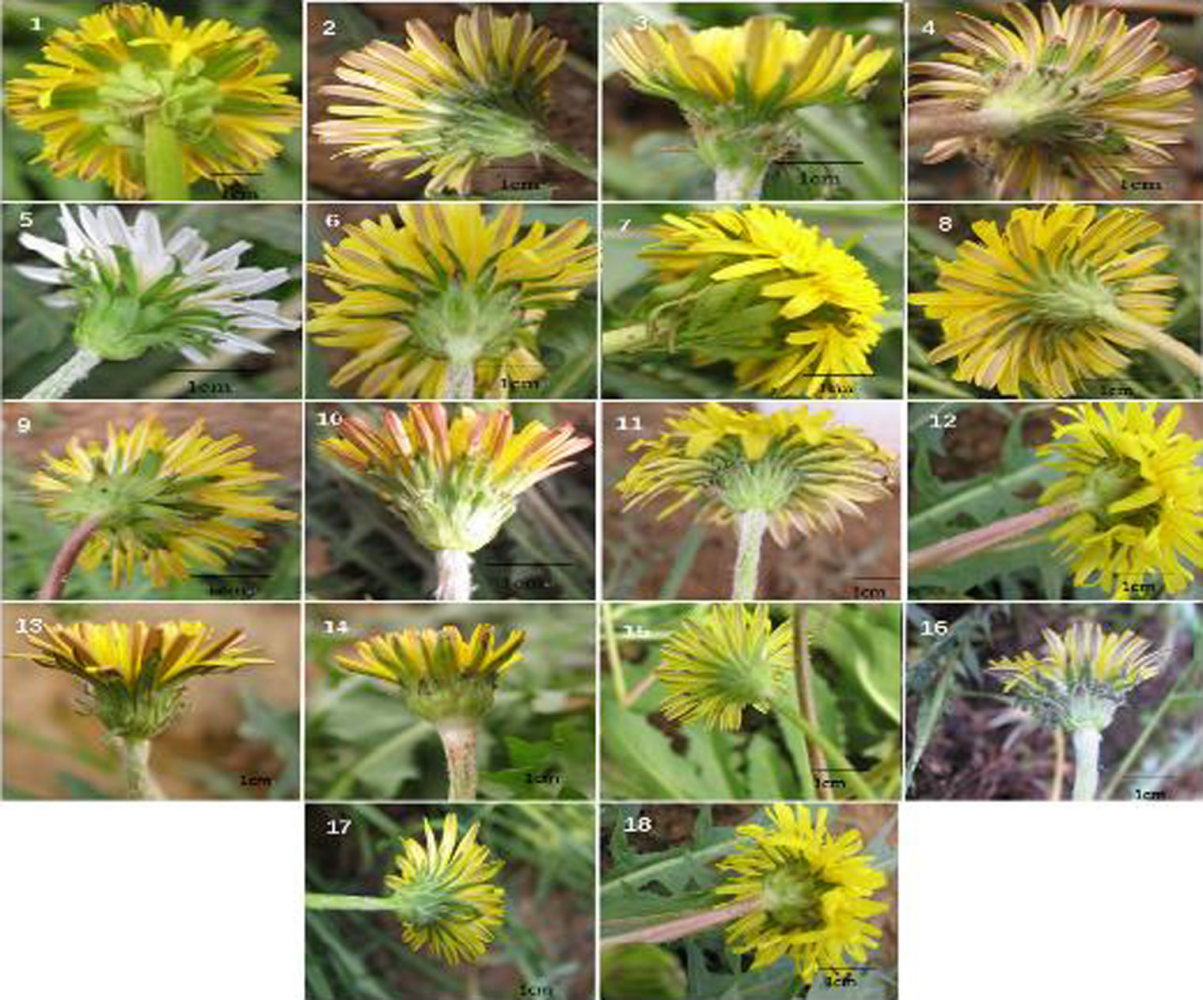
Pictures of flower organ of the 18 taxa of northeast China native *Taraxacum* species

**FIGURE 3:**
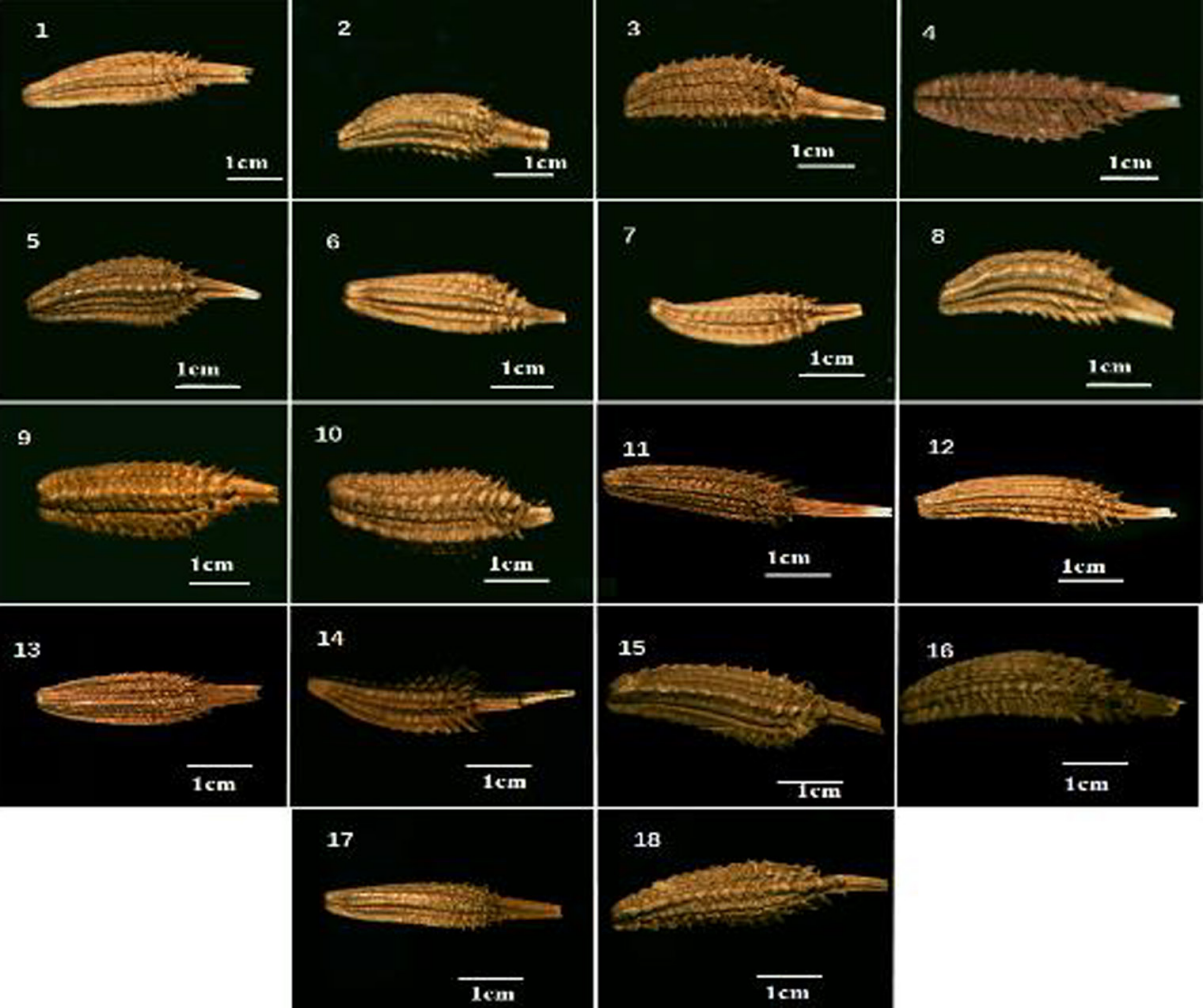
Achene of the 18 taxa of northeast China native *Taraxacum* species

Thirty‐two morphological characteristics were evaluated (Table 2). The plant height was divided into three categories: short (5–10 cm), medium (10–15 cm), and tall (15–20 cm). The leaf length and width ranged from 6 cm to 20 cm and from 1.2 cm to 2.9 cm, respectively (Table 3). The leaf thickness ranged from 0.61 mm to 1.26 mm. The leaf anatomy in cross‐section and floral and achene traits differed among the 18 taxa, with pubescent leaves compared with thicker, glabrous leaves in other species (Figures 1, 2, 3). Three types of leaf margin were observed: deeply pinnatifid (pinnatisect), moderately pinnatifid (pinnatifid to pinnatisect), and shallowly pinnatifid. The apical lobe shape of the leaves was primarily triangular, but the shapes ranged from triangular, triangular‐oblong, diamond‐shaped triangle, to triangular‐hastate (Table 2). In *T*. *asiaticum*, *T. coreanum*, *T*. *ohwianum*, *T. erythropodium*, and *T*. *formosanum*, the margins of the middle leaves had lateral lobes. *T. coreanum* had white flowers, while the other species had yellow flowers (Figure 2). The involucres were described in terms of the pubescence, arrangement, and structure of the outer and inner phyllaries. The outer phyllaries were ovate‐lanceolate, lanceolate, or ovate (Table 2 and Figure 2). A minority of the outer bracts were lanceolate, most of the outer bracts were ovoid or linear‐lanceolate, most of the inner bracts were lanceolate, and the inner bracts were corniculate. The scape pubescence was categorized as dense, sparse, or lacking (scape glabrous).

**TABLE 2:** Mean values of 32 horticultural qualitative and quantitative traits in the 18 taxa of northeast China native *Taraxacum* species used for multivariate analysis

Species^a^	Plant	Leaf						
	Plant height (cm)	Leaf length (cm)	Leaf width (cm)	Leaf thickness (mm)	Hair on leaf^b^	Leaf margin	Apical lobe shape	Lateral lobes
1	12.71	10.21	1.71	0.61	−	1	2	−
2	6.11	5.42	1.32	0.65	+	2	1	+
3	8.21	7.31	2.11	0.69	−	1	1	+
4	8.12	11.23	1.32	1.04	+	1	2	−
5	8.51	7.61	0.81	1.26	+	2	3	+
6	19.41	14.41	2.91	1.13	+	2	4	−
7	12.61	11.42	1.62	0.61	−	1	3	−
8	10.92	7.21	1.53	0.75	+	1	1	+
9	11.81	10.41	1.71	1.03	+	2	1	−
10	12.73	11.71	1.22	1.05	+	3	2	−
11	10.21	15.32	0.94	0.75	−	3	1	+
12	8.22	7.33	3.41	1.02	−	1	1	−
13	11.33	17.21	1.13	0.84	−	1	2	+
14	8.61	9.51	2.81	1.12	+	3	3	−
15	7.62	8.42	1.82	0.62	+	1	2	−
16	12.91	18.51	3.23	1.78	−	1	1	+
17	11.81	18.42	0.71	0.42	−	1	1	+
18	6.71	8.71	1.12	1.08	+	2	2	−

Species	Inner bracts		Scape			Pollen			
	Inner bracts shape	Corniculate	S/L ratio^c^	Pubescence on scape	Color of scape base	Length of the polar axis (μm)^d^	Equatorial axis (μm)^d^	*P*/*E* ratio	Pollen shaped
1	1	−	0.7	1	1	19.81–22.32	16.74–18.41	1.19	1
2	1	+	1.2	2	1	21.93–24.84	20.63–22.76	1.06	2
3	1	+	1.1	3	1	20.51–22.75	18.72–20.33	1.09	2
4	1	+	1.2	2	2	19.97–22.16	17.25–17.91	1.16	1
5	1	+	1	3	1	30.53–31.21	22.56–23.44	1.35	3
6	2	−	1.2	3	1	18.95–20.54	16.57–17.95	1.14	1
7	1	−	1	1	1	20.86–23.53	17.93–19.66	1.16	1
8	3	+	1	1	1	26.32–27.15	22.51–24.18	1.17	1
9	1	+	1.1	1	2	23.41–24.68	19.96–21.62	1.07	1
10	3	+	1	3	1	24.23–25.27	21.76–22.31	1.11	2
11	1	+	1	2	1	21.43–23.25	17.02–18.53	1.26	2
12	1	−	0.8	1	2	20.07–22.08	17.28–18.02	1.16	1
13	1	+	1	2	1	20.43–21.21	21.21–22.34	0.96	1
14	2	−	0.8	1	1	19.05–20.14	18.54–19.35	1.03	1
15	3	+	1	3	1	20.46–21.53	18.73–19.26	1.09	1
16	2	−	1	3	1	26.12–27.05	22.31–23.78	1.17	2
17	2	−	1.2	2	1	20.42–21.68	19.66–20.82	1.04	2
18	2	+	1	2	2	20.23–21.27	19.56–20.21	1.03	1

Species	Achene						
	Color of achene	Achene length (cm)	Achene width (cm)	Beak length (cm)	BL/AL ratio^e^	Achene shape	Pappus
1	1	2.91–3.22	0.55–0.57	0.60–0.62	0.21	1	−
2	1	3.01–3.22	0.71–0.93	1.02–1.04	0.34	1	−
3	1	3.83–4.21	1.33–1.62	2.41–2.43	0.63	2	+
4	3	4.41–4.71	1.32–1.51	1.07–1.09	0.24	2	−
5	2	3.82–4.23	1.55–1.60	1.16–1.19	0.30	1	−
6	2	3.01–3.52	0.85–0.92	1.08–1.11	0.36	1	−
7	1	2.85–2.92	0.55–0.60	0.52–0.54	0.18	1	−
8	2	3.13–3.42	0.81–1.09	1.16–1.19	0.37	1	−
9	3	4.42–4.82	1.24–1.53	1.11–1.14	0.25	3	−
10	3	4.32–4.71	1.12–1.25	1.04–1.06	0.24	4	−
11	2	4.03–4.21	1.33–1.62	2.78–2.93	0.69	1	+
12	2	3.41–3.71	1.31–1.41	1.27–1.29	0.37	1	+
13	2	3.74–3.81	1.38–1.42	1.16–1.19	0.31	1	−
14	1	3.31–3.42	0.86–0.91	2.08–2.11	0.63	1	+
15	1	2.88–2.95	1.85–1.87	1.16–1.19	0.41	1	−
16	1	3.23–3.35	1.81–1.89	0.82–0.84	0.26	1	−
17	3	2.94–3.05	0.84–0.88	1.11–1.14	0.38	1	−
18	3	3.05–3.11	1.12–1.15	1.14–1.16	0.37	1	−

Note

Explanatory comment: Leaf margin of Deep “1”; Medium “2”; Shallow “3”; Triangle of triangular‐hastate “1”; Triangle “2”; Triangular‐oblong “3”; Diamond‐shaped triangle “4.”

Explanatory comment: inner bracts shape of Lanceolate “1”; Ovate‐lanceolate “2”; Linear “3”; pubescence on scape of glabrous “1”; sparsely “2”; densely “3”; color of scape base of green “1”; purple “2”; pollen shape of subprolate “1”; spheroidal “2”; prolate “3.”

Explanatory comment: color of achene of Light brown “1”; Brown “2”; Dark brown “3”; Achene shape of Lanceolate‐oblong “1”; Lanceolate‐obovate “2”; Obovate‐oblong “3”; Narrowly obovate “4.”

^a^ A, *T. antungense* Kitag.; B, *T. asiaticum* Dahlst.; C, *T. variegatum* Kitag.; D, *T. mongolicum* Hand.‐Mazz.; E, *T. coreanum* Nakai.; F, *T. ohwianum* Kitag.; G, *T. urbanum* Kitag.; H, *T. asiaticum var. lonchophyllum* Kitag.; I, *T. formosanum* Kitam.; J, *T. liaotungense* Kitag.; K, *T. sinomongolicum* Kitag.; L, *T. heterolepis* Nakai et Koidz. ex Kitag.; M, *T. brassicae folium* Kitag.*;* N, *T. platypecidum* Diels*;* O, *T. falcilobum* Kitag.; P, *T.borealisinense* Kitam.; Q, *T. junpeianum* Kitam.; R, *T. erythopodium* Kitag.

^b^ “–” means does not exist and “+” means for exist.

^c^ S/L ratio: scape length/leaf length ratio.

^d^ Pollen shape: spheroidal = 0.88–1.14, subprolate = 1.14–1.33, prolate = 1.33–2.00.

^e^ BL/AL ratio means the beak length/achene length.

**TABLE 3:** Principal component analysis (PCA) of 32 morphological characteristics in the 18 taxa *Taraxacum* species of northeast China

Total variance explained						
Component	Initial eigenvalues			Extraction sums of squared loadings		
	Total	% of variance	Cumulative %	Total	% of variance	Cumulative %
1	9.483	29.635	29.635	9.483	29.635	29.635
2	5.566	17.393	47.029	5.566	17.393	47.029
3	4.594	14.356	61.385	4.594	14.356	61.385
4	3.485	10.892	72.277	3.485	10.892	72.277
5	2.802	8.755	81.032	2.802	8.755	81.032
6	2.167	6.771	87.803	2.167	6.771	87.803
7	1.535	4.798	92.602	1.535	4.798	92.602
8	1.316	4.113	96.715	1.316	4.113	96.715
9	1.051	3.285	100	1.051	3.285	100

Note

Extraction method: Principal component analysis.

**FIGURE 4:**
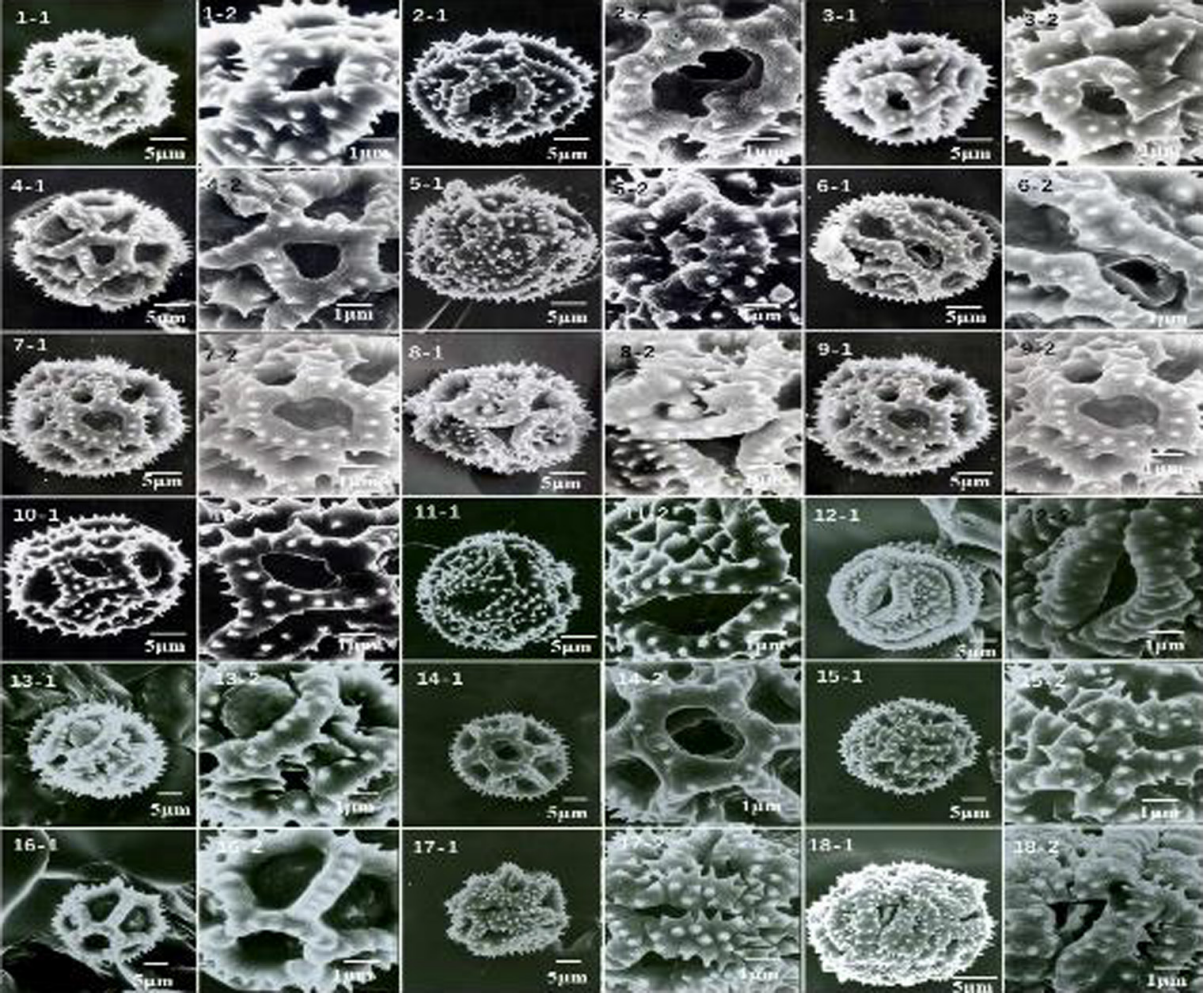
Micrographs of pollen grains in 18 *Taraxacum* species, examined by scanning electron microscopy (SEM)

The achenes of dandelions generally consist of three parts: the achenes body, the achene beak base, and pappus. The survey revealed some common features in achenes’ micromorphology: the achenes were covered entirely with spines, which were classified into small or large spines, and further into large spines, small dense spines, and small sparse spines. Common achene micromorphological features included an achene body with spines and spines with white spots; spines arranged in regular rows formed a higher consistency (Table 2 and Figure 3). The color of the achenes varied from light brown to brown to dark brown and was crimson. The longest achenes were 4.41–4.82 mm and the shortest were 2.85–2.92 mm. The achene shapes were lanceolate‐obovate, obovate‐oblong, narrowly obovate, and lanceolate‐oblong in other species. The smallest achenes were present at 2.88 cm × 1.85 cm and the largest at 4.82 cm × 1.24 cm (Table 3 and Figure 4). The longest achene beak measured 2.78–2.93 cm. The achene length to beak length ratio (BL/AL) was the highest at 0.69 and the lowest at 0.18. The size and micromorphological characteristics of the achenes were relatively stable within a species, but were significantly different among the species. However, shallow and deep depressions observed on the achene surface are unstable features with little significance in the identification of *Taraxacum* species.

Pollen from 18 taxa was observed by light microscopy and SEM. The pollen morphological structures of the 18 *Taraxacum* species had conspicuous characteristics of *Compositae* pollen; namely, the pollen was nearly spherical, with three indehiscent ditches and a spiny surface. Pollen had the longest polar axis (P) at 30.53–31.21 μm and the shortest at 18.95–20.54 μm (Table 2 and Figure 4). Pollen varied from spheroidal to subprolate or prolate. The pollen of the 18 species was prolate to spheroidal (*P*/*E* = 0.95–1.36). The *P*/*E* max ratio was 1.36 (prolate); the *P*/*E* min ratio was 0.96 (subprolate). In the study of palynology, pollen size, pollen shape, type of germ pore, and exine patterns were specific to the species, thus suitable for the delimitation of *Taraxacum* species growing in northeastern China. The data characteristics of pollen size, spine width, spine density, germination pore size, and pore size can be used as an auxiliary basis for the classification of *Taraxacum*.

### 3.2 Taxonomic relationships inferred from morphological characteristics based on PCA and cluster analysis

**FIGURE 5:**
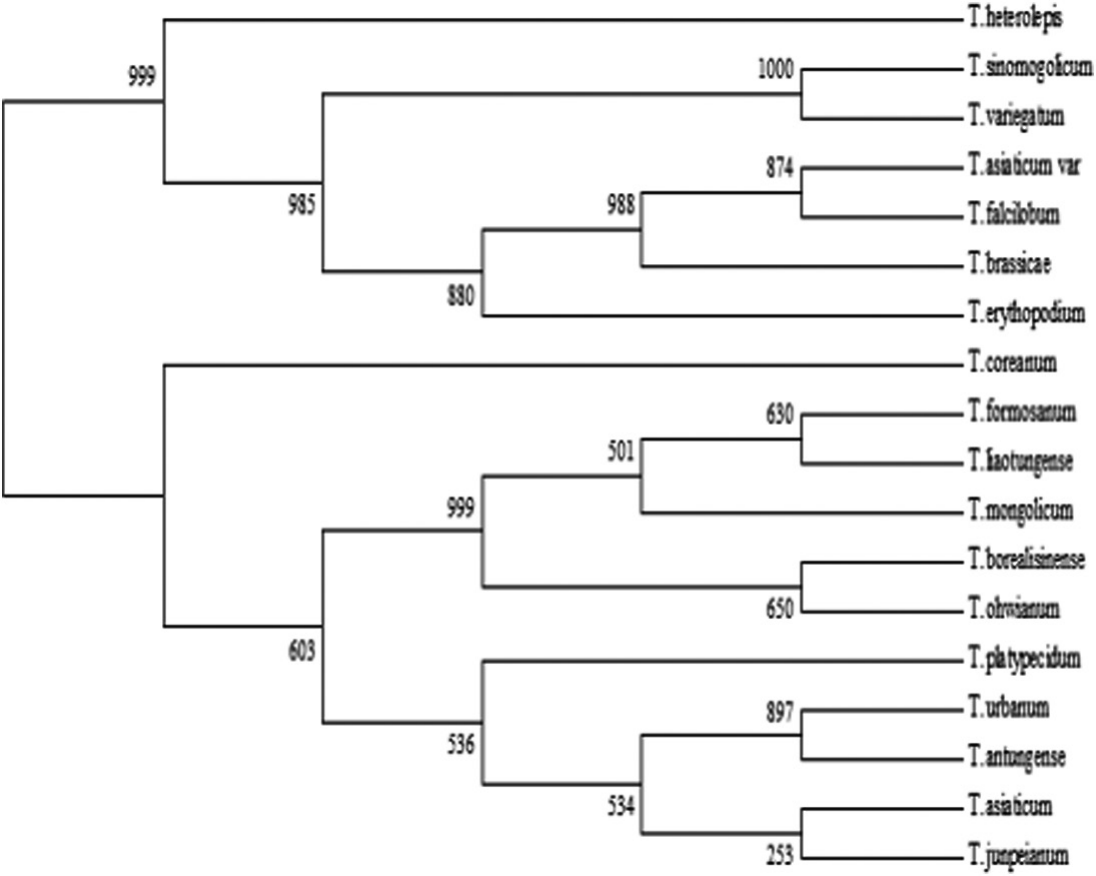
Dendrogram of grouping based on morphological characteristics of the 18 taxa of northeast China native *Taraxacum* species by the cluster analysis using average linkage (between groups)

Thirty‐two qualitative and quantitative morphological traits were subjected to multivariate analysis. Of these, 21 variables were significantly different among the taxa. PCA returned nine principal components that explained 96.7% of the variation (Table 3). The eigenvalues of each principal component indicated that the first, second, and third principal components were associated with 21, 6, and 4 variables, respectively, of the 32 morphological characteristics. Three major principal components covered 31 characteristics and explained 61.4% of the results. The first principal component (PC 1) was associated with 21 morphological characteristics, including the plant height, leaf length, leaf width, BL/AL, leaf thickness, leaf pubescence, abaxial corolla background color, abaxial corolla stripe color, membranous, corniculate flower, outer bracts shape, inner bracts shape, and corniculate. Cluster analysis using the average linkage (between groups) of the standardized first three principal components produced three groups (Figure 5). Group I included one species *T*. *coreanum* with white flowers, indicating the contribution of flower color to the classification. Group II comprised seven species: *T. heterolepis*, *T. sinomongolicum*, *T*. *variegatum*, *T. asiaticum* var*. lonchophyllum*, *T. falcilobum*, *T. brassicaefolium*, and *T. erythropodium* (outer involucre bracts, narrow membranous or nonmembranous). Group III included 10 species: *T. formosanum*, *T*. *liaotungense*, *T*. *mongolicum*, *T. borealisinense*, *T*. *ohwianum*, *T. platypecidum*, *T*. *urbanum*, *T. antungense*, *T*. *asiaticum*, and *T. junpeianum* (outer involucre bracts, broad membranous).

### 3.3 ITS sequence analysis

General ITS sequence primers were used to amplify *Taraxacum*. Most of the materials showed bright and clear bands around 730 bp, and the amplification products were between 750 bp and 500 bp, all of which were recovered, cloned, and sequenced using T vector. The length of the ITS region ranged from 728 bp to 733 bp. The aligned ITS matrix was 733 bp long, the ITS‐1 region was 276 bp, the ITS‐2 region was 295 bp, and the 5.8S recombinant DNA (rDNA) sequence was 162 bp long. A BLAST search of all sequences showed 99% similarity to a partial sequence of the ITS region in *T. coreanum* (accession number JF837599.1). Of these, 162 (22%) were constant and 569 (78%) were potentially informative. The consistency index of the maximum parsimonious tree was 0.051 and the retention index was 0.007. The ITS‐based topology of the 18 species was consistent with the morphological characteristics of plants. The total G + C content varied among the taxa, from 51.23% in *T*. *ohwianum* to 52.53% in *T. coreanum*. Several base variation sites were identified in *Taraxacum*, and the information sites were approximately 3–4. The pairwise difference of the ITS base composition between the 18 taxa was within 1%.

## 4 DISCUSSION

### 4.1 Evolution trend in *Taraxacum* species

The taxonomy of the *Taraxacum* complex group is controversial, owing to the lack of available criteria to evaluate the systematics of these species. The taxonomic and systematic ranking of these species is doubtful, owing to their complex morphological characteristics and molecular evidence (Li & Chen, 2013). Morphological classification is the basis of plant taxonomy. Based on the morphological classification and assisted by palynology, chemistry, and ITS sequence analysis, we classified and identified 18 *Taraxacum* species in northeast China. Both qualitative and quantitative characteristics found in some of the newly reported species were consistent and similar to those found in previous studies (Yamaji et al., 2007). In the “*Flora of China*,” the floral organs of dandelion were recorded as the main basis for morphological classification. In the present study, we found that the achenes’ characteristics of the dandelions can also be used as the basis of classification to identify species useful in traditional Chinese medicine. Achenes, as reproductive organs, are in a relatively enclosed environment, are less affected by external environmental factors than floral organs, and harbor stable genetic traits (Ning et al., 2012; Wu et al., 2011). This trait in achene micromorphology may provide new evidence for phylogenetic studies of *Taraxacum*. The micromorphological characteristics of *Taraxacum* species examined herein, which included a whole achene spiny body, are complex and belong to a relatively evolutionary group. Compared with the results of previous studies, the main evolutionary features of *Taraxacum* are achenes with a short and thick beak that is not obvious, and an achene wall without strumae or small spines, or partially or completely covered with tumor or spines (Lee et al., 2011). Therefore, the achene morphology indicates that *Taraxacum* is a well‐evolved genus. It is worth mentioning that the BL/AL ratio, which is a fixed value in the same species of *Taraxacum*, indicates that beak base size and achene length are quality traits that are not easily affected by the environment and stable genetic characters, so they can be used as the basis for species classification.

The results of the ITS sequence analysis were supported by the morphological traits of the sampled taxa. Eighteen taxa from northeastern China were divided into three groups. The taxa within Group II were characterized by closely arranged outer bracts with hornlike protuberances, large achenes shaped like spindles or inverted cones, and covered with spines. Group III was characterized by outer involucres basally unrolled or rolled, outer bracts without hornlike projections, smaller achenes, and an achene lower half with tuberous projections. The results of this study support the taxonomic classification of this genus in the “*Flora of China*.”

### 4.2 The causes for the complicated nature of *Taraxacum* classification

The taxa examined during the eight years of the study under different environmental conditions differed in terms of their leaf, flower, inflorescence, pollen, and achene morphology (Wang et al., 2010). Of the 32 analyzed morphological characteristics, 31 exhibited significant differences among the taxa. The PCA and cluster analyses of those characteristics arranged the taxa into three groups. In particular, the BL/AL ratio of the achene morphological characteristics was not used for species delimitation in the “*Flora of China*”; the results of our study suggest that this character is potentially useful in the identification of *Taraxacum* species.

Several reasons for the difficulty in classifying *Taraxacum* can be identified based on the results of this study. The first reason is that there are significant intraspecific variations in *Taraxacum* species. Owing to the high plasticity of the genus, it is found throughout China, resulting in high species and intraspecific variations that hinder their classification. The second reason is that apomixis, one of the relatively primitive reproductive modes, contributes to the geographic isolation of *Taraxacum* species, while the lack of gene exchange between populations of the same species impedes the delimitation of biological species. The third reason is that traditional taxonomic evidence other than morphology, such as palynology, cytology, embryology, chemistry, and molecular biology alone, are insufficient for species identification (Kumar et al., 2015; Thompson et al., 1997). The palynological evidence presented herein suggest that pollen morphological characteristics and clustering analyses support the treatment of *Taraxacum* species from the northeastern China as presented in the “*Flora of China*.” Finally, different authors adopt different interpretations of the species concept and species range of distribution, resulting in controversy.

## 5 CONCLUSIONS

Dandelion plants, which belong to the *Compositae* family, constitute one of the most evolutionarily diverse subfamilies used in Chinese medicine and are widely distributed in China. In this study, we surveyed most regions in China. However, dandelion classification was difficult because species boundaries are often confused. In this study, we completed the dandelion germplasm resource classification and evaluation, which can supplement and perfect the “*Flora of China*” dandelion classification key points. Our results will clarify the distribution of species, facilitating the development of dandelion medicinal resources, determination of medicinal value, and identification of potential medicinal species for use in traditional Chinese medicine.

## CONFLICT OF INTEREST

There is no conflict to declare.

## AUTHOR CONTRIBUTIONS

**Wu Jie:** Conceptualization (lead); Funding acquisition (lead); Methodology (lead); Project administration (lead); Resources (lead); Validation (lead); Writing – original draft (lead). **Liu Qun:** Formal analysis (supporting); Resources (supporting); Software (supporting). **Haitao Cheng:** Formal analysis (supporting); Validation (supporting). **Wei Ning:** Conceptualization (equal); Visualization (supporting). **Wei Cao:** Investigation (supporting); Writing – review & editing (supporting).

## ETHICAL APPROVAL

This research does not involve any studies with human and animal testing.

## Data Availability

The data that support the findings of this study are available on request from the corresponding author. The data are not publicly available due to privacy or ethical restrictions.
